# Systems Biology in Cancer Diagnosis Integrating Omics Technologies and Artificial Intelligence to Support Physician Decision Making

**DOI:** 10.3390/jpm13111590

**Published:** 2023-11-10

**Authors:** Alaa Fawaz, Alessandra Ferraresi, Ciro Isidoro

**Affiliations:** Laboratory of Molecular Pathology, Department of Health Sciences, Università del Piemonte Orientale, 28100 Novara, Italy; 20041632@studenti.uniupo.it (A.F.); alessandra.ferraresi@med.uniupo.it (A.F.)

**Keywords:** artificial intelligence, medical technology, smart health, digital health, omics technologies, imaging, diagnosis, personalized medicine

## Abstract

Cancer is the second major cause of disease-related death worldwide, and its accurate early diagnosis and therapeutic intervention are fundamental for saving the patient’s life. Cancer, as a complex and heterogeneous disorder, results from the disruption and alteration of a wide variety of biological entities, including genes, proteins, mRNAs, miRNAs, and metabolites, that eventually emerge as clinical symptoms. Traditionally, diagnosis is based on clinical examination, blood tests for biomarkers, the histopathology of a biopsy, and imaging (MRI, CT, PET, and US). Additionally, omics biotechnologies help to further characterize the genome, metabolome, microbiome traits of the patient that could have an impact on the prognosis and patient’s response to the therapy. The integration of all these data relies on gathering of several experts and may require considerable time, and, unfortunately, it is not without the risk of error in the interpretation and therefore in the decision. Systems biology algorithms exploit Artificial Intelligence (AI) combined with omics technologies to perform a rapid and accurate analysis and integration of patient’s big data, and support the physician in making diagnosis and tailoring the most appropriate therapeutic intervention. However, AI is not free from possible diagnostic and prognostic errors in the interpretation of images or biochemical–clinical data. Here, we first describe the methods used by systems biology for combining AI with omics and then discuss the potential, challenges, limitations, and critical issues in using AI in cancer research.

## 1. Introduction

Delayed diagnoses, misdiagnoses, and missed diagnoses impact patient health and safety, and have great societal consequences. Mistakes in diagnosis may account for up to 60% of all medical errors and are accountable for up to 80,000 deaths in U.S. medical centers each year [1]. Typically, clinicians have limited time to make decisions based on the interpretation of huge amounts of laboratory, imaging, and clinical data, and this increases the risk of underestimating (or sometimes overestimating) some data. Furthermore, subjective factors, such as personal experience and medical specialty, are potential bias factors that influence the accuracy of diagnosis [2].

Artificial Intelligence (AI), a field of computer science used for prediction and automation, has emerged as a potential solution to promote a precision approach in healthcare and is expected to reduce errors caused by human judgment in various medical domains [3].

Cancer is the leading cause of death in people, accounting for an estimated 10 million deaths by 2020 [4]. It is a complex disease resulting from anomalies in physiological processes involving genes, coding and non-coding RNAs, proteins, metabolites, and other biomolecules [5,6]. To understand such a complex disease from its onset to its progression, multi-omics analysis of these numerous bio-entities is required. Modern biotechnologies allow for the high throughput analysis of the sequence and expression of many genes (genomics and epigenomics), proteins and their post-translational modifications (proteomics, phospho-proteomics and glycol-proteomics), RNAs (RNA transcriptomics), non-coding RNAs (including miRNAs and long-non-coding RNAs), and metabolites (metabolomics) from the same organism [7]. However, a platform where all these big data are integrated to uncover correlations and synergisms among the biological pathways and processes is required. Systems biology combines the power of AI and of multi-omics technologies for modeling the signaling and metabolic signature of a given cancer. This is instrumental for designing effective diagnostic and prognostic markers and novel and patient-tailored therapeutic interventions.

Despite difficulties in providing individualized and data-driven care, advancements in screening, diagnosis, treatment, and survival rate in cancer patients have been remarkable in recent decades [8]. Early detection and prognosis prediction represent two crucial clinical needs for limiting cancer progression. Body and organ computed scan methodologies, the histopathology imaging of biopsies, and a range of blood tests for detecting biomarkers are instrumental in the initial diagnosis process and for determining cancer staging, the grade of malignancy, and prognosis. These approaches do not provide information on the molecular alterations that precede and follow the onset of cancer. Molecular and omics technologies can provide a genetic, epigenetic, and metabolic profile of the tumor that can better define such alterations thus helping to determine the most appropriate treatment as well as predict the response to therapy [9,10].

The development and extensive use of high-throughput technologies has ushered in the era of biological and medical big data. This has led to the accumulation of data sets on a large scale, thereby opening a wide range of potential applications for data-driven methods in cancer treatment, spanning from basic research to clinical practice: molecular tumor characterization, tumor heterogeneity, drug discovery and potential therapeutic strategies. As a result, the data-driven research field of bioinformatics adapts data mining techniques, such as systems biology, machine learning, and deep learning, which are discussed in this review paper. Systems biology uses a data-driven approach to identify important signaling pathways. The pathway-oriented analysis is extremely important in cancer research because it helps researchers comprehend the molecular features and heterogeneity of tumors and tumor subtypes [11]. In this context, the proper clinical care for cancer patients can be improved by the introduction of AI in cancer detection, diagnosis, and treatment [12,13,14,15].

AI-based technologies applied to oncology aim at improving clinical practice, including but not limited to the early and accurate diagnosis and prediction of personalized outcomes (i.e., prognosis and therapy response), by acquiring a profound perception of tumor molecular biology through the association of multiple biological parameters [16].

### Artificial Intelligence in Medicine at Glance

**Figure 1:**
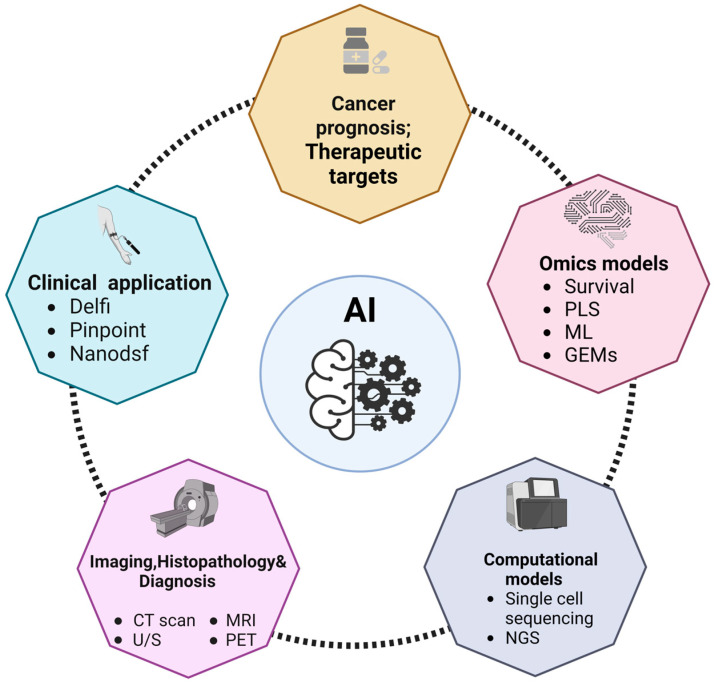
Overview of the applications of AI to cancer diagnosis and oncology research field. The scheme depicts the main fields of application of AI discussed in this review. Abbreviations: computed tomography, CT; gene expression models, GEMs; machine learning, ML; magnetic resonance imaging, MRI; nano differential scanning fluorimetry, Nanodsf; next-generation sequencing, NGS; positron emission tomography, PET; partial least squares analysis, PLS; ultrasound imaging, U/S.

AI is meant to mimic human cognitive abilities in elaborating the information but at a much higher speed and with no emotional interference. The main types of AI that apply to cancer-patient healthcare include machine learning (ML) and its evolved subtype deep learning (DL), which can assist in making a rapid and more accurate diagnosis (based on biochemical, clinical data, and medical imaging), in discovering and developing new drugs, in designing personalized therapy, in predicting the therapy response, and in guiding the robotic surgery [17,18] (Figure 1).

Current AI systems have been involved to be used in a variety of clinical settings, including (i) image-based computer-aided discovery and diagnosis in various medical specialties, (ii) the translation of genomic information for recognizing genetic variants using high-throughput sequencing technologies, and (iii) the prediction and tracking of patient’s prognosis [19,20]. Moreover, they have been implemented as well in (iv) the discovery of new biomarkers by combining omics and phenotype data, (v) the detection of health status using biological signals (e.g., enzyme activity and protein concentration) obtained from wearable devices, and (vi) the production and implementation of autonomous robots in medical procedures [19,20].

The creation of AI models that predict the properties of vast and interconnected networks found in living organisms would allow for a thorough examination of how signaling molecules generate functional cellular reactions. Machine learning (ML) algorithms, a subset of AI, are capable of making decisive interpretations of large, complex data sets, making them an effective tool for analyzing and comprehending multi-omics data for patient-specific observations [20]. We can anticipate the remarkable growth of AI in the medical field in light of the digital acquisition of high-dimensional and annotated medical data, the progress of ML methods, open ML data science, and advancements in computational power and storage services [20]. AI is expected to make it easier to diagnose specific illnesses in patients. Commonly, deep learning (DL) architectures are analogous to artificial neural networks of multiple non-linear tiers. Over the past decade, a large variety of DL designs have been developed depending on the input data type and the purpose of the research. Moreover, the assessment of the model’s efficiency has revealed that DL application on cancer prognosis surpasses other traditional ML techniques. DL frameworks have also been used in cancer diagnosis, classification, and treatment by utilizing genomic profiles and phenotype information. Systems biology has been an effective method to comprehend the complex molecular profile of cancers, interpret the mechanisms of tumor progression, and allow for the amalgamation of omics data as well as the characterization of diverse tumors [21,22].

## 2. Omics Data for Identifying Cancer Metabolic Biomarkers

Omics technologies allow for the in depth analysis of the molecular characteristics of cancer at both bulk and single-cell level, providing a wealth of multi-omics data that challenge the capability of scientists and medical doctor to combine for drawing a consistent picture of the multilayer complexity of cancer biology. Genomic, epigenomic, transcriptomic, proteomic, and metabolomic data can be elaborated using appropriate models for making predictions about prognosis and treatment response in a patient-tailored (personalized) manner [13,15,22].

### 2.1. Survival Models

**Figure 2:**
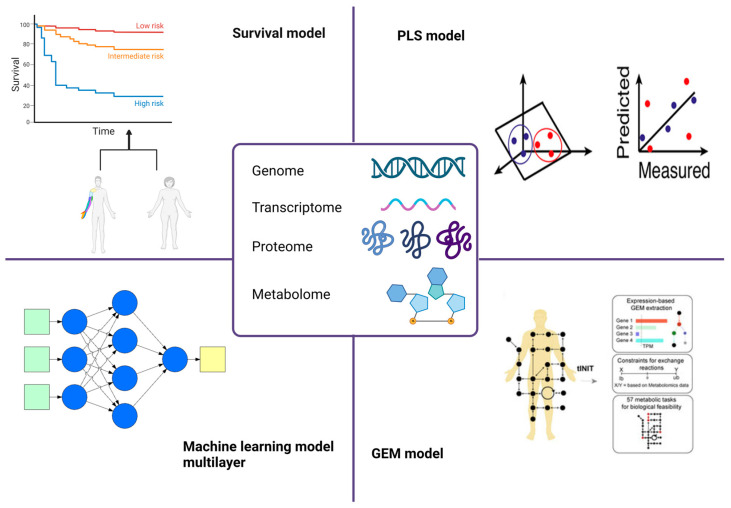
Overview of the omics technologies exploited in cancer diagnosis/prognosis. The scheme depicts the main omics models currently used in biomarker identification. Abbreviations: gene expression modeling, GEM; partial least squares analysis, PLS.

To find cancer metabolic biomarkers, survival models have been used more frequently than partial least squares (PLS) models, ML models, and gene expression modeling (GEM) [23] (Figure 2). The Kaplan–Meier method, the log-rank test, and/or the Cox regression model are representative survival models used in cancer studies. These models are used to describe the likelihood of survival (or survival curve) for a group of patients after treatment, compare the survival curves of two or more treatment groups, and describe the effects of multiple explanatory (independent) variables, profiles of gene expression, and metabolite concentration) on survival curves, respectively. In contrast to Kaplan–Meier models, which must discretize their data, the Cox regression model has the advantage of processing continuous values directly, minimizing data loss [24]. In their study, based on GEM of seven major metabolic pathways, Peng and colleagues identified 30 tumor subtypes in 33 different cancer types (such as breast invasive carcinoma, cholangiocarcinoma, colorectal cancer, glioblastoma multiforme, gastrointestinal tumors, lung cancer, pancreatic cancer, and ovarian serous cystadenocarcinoma, among others) and evaluated the clinical utility of so-called metabolic expression subtypes. For this, correlations between metabolic expression subtypes and their corresponding prognosis were investigated using the Kaplan–Meier method, log-rank test, and Cox regression model. Consequently, subtypes with upregulated lipid metabolism appeared to have a better prognosis than subtypes with upregulated glycemic, nucleotide, vitamin, and cofactor metabolism. The association of various somatic mutations in cancer driver genes with metabolic expression subtypes has also been discovered. Two transcription factors, SNAI1 and RUNX1, were identified from knockdown studies as potential therapeutic targets for a subtype of cancer with upregulated carbohydrate metabolism that consistently had a poor prognosis across cancer types [23].

### 2.2. PLS Models

Partial least squares regression (PLS) was initially created as a regression model that processes numerous independent variables that are correlated and produce numerous dependent variables, which many statistical and ML techniques cannot directly handle. PLS models and their variations, particularly PLS-discriminant analysis (PLS-DA) are frequently used for the analysis of omics data with a focus on metabolomics [25]. PLS-DA has been primarily used to extract insights from large datasets of omics data, such as identifying metabolites from metabolome data that differentiate between cancer cells in their various statuses. PLS-DA might have an overfitting issue too, like other data mining techniques, so it needs thorough validation, frequently performed through cross-validation [26].

PLS-DA and its variants have been used to analyze metabolome data to identify a variety of cancers, including breast cancer, glioma, non-small cell lung cancer, oral precancerous cells, cervical precancerous lesions, and prostate cancer [27,28]. Among its advantages, PLS-DA allows for the analysis of highly collinear and noisy data. Moreover, the calibration model provides a subset of useful statistics, including prediction accuracy, scores and loading plots. However, a potential limitation has emerged when this method was applied to metabolomics; the use of this model by non-experts may produce inaccurate results, owing to a lack of appropriate statistical validation [29] (Table 1).

### 2.3. Genome-Scale Metabolic Models

Gene expression modeling (GEM) is a computational model based on the law of mass conservation of metabolites and allows for the prediction of metabolic fluxes for entire biochemical reactions taking place inside a cell by using numerical optimization [30,31]. Technically, GEM describes the participation of each metabolite for an entire set of biochemical reactions in the form of a stoichiometric matrix and is simulated using varied forms of objective functions and constraints that reflect genetic and environmental conditions of interest. As a result, GEM allows for the efficient simulation of a target cell’s metabolic phenotypes under a wide range of genetic and environmental conditions. GEM can also be integrated with omics data, such as RNA-seq, for building a cell-specific model and thereafter modeling multicellular organisms. In comparison with ML models, GEMs generate more interpretable prediction outcomes that grasp a cell-specific metabolic phenotype. GEM simulations, however, demand consideration. Due to the possibility of biologically incorrect objective functions or constraints, it is advised to proceed with the analysis of the predicted intracellular metabolic flux distributions from GEMs with caution. A representative issue is the use of constraints that do not accurately reflect a culture medium. Finally, GEMs do not directly produce additional data for regulatory and signaling networks, which are also crucial for understanding the physiology of a cell [32,33] (Table 2).

### 2.4. Machine Learning Models

The classification task of disease prediction has been thoroughly studied in medical oncology and cancer research, based on well-established machine learning algorithms for dealing with binary or multi-class learning problems. Patient categorization would allow for the development of ML-based predictive models capable of assessing risk stratification with generalizable performance. Based on images and genetic data, DL models were trained to classify and detect disease subtypes. These data-driven approaches demonstrated the superiority of ML-based frameworks for leveraging heterogeneous datasets for improved diagnosis and treatment [34].

### 2.5. Deep Neural Networks (DNNs)

Deep neural network (DNN) models are rapidly evolving and becoming more sophisticated. They have been widely used in biomedical research across the board. Initially, large-scale imaging and video data aided its development. While most biomedical data sets are not considered big data, the rapid data accumulation enabled by NGS made it suitable for the application of DNN models that require a large amount of training data [35]. In 2019, for example, Samiei et al. used TCGA-based large-scale cancer data as benchmark datasets for bioinformatics machine learning research, such as Image-Net in computer vision [36]. Following that, large-scale public cancer data sets like the TCGA encouraged the widespread use of DNNs in cancer research [37] (Table 3).

### 2.6. Graph Neural Networks (GNNs)

Graph neural networks (GNNs) have achieved great results and are being progressively employed in a node classification task. It offers a strategy to acquire novel representations of nodes by combining the features of its local neighborhood and connectivity. Recently, some GNN-based approaches have been proposed to forecast the molecular subtyping of cancer. Rhee et al. created a graph convolutional network (GCN)-based model to investigate the gene–gene alliance and information transmission for cancer subtyping [38]. Lee et al. developed a GCN model with a focus on the mechanisms to learn pathway-level representations of cancer samples for their subtype classification [39]. Even though GNNs are strong, it is reported that they are susceptible when the structure of the graph and nodes’ features are polluted with noise [40]. Thus, a robust GNN model is required for the precise and stable prediction of cancer subtypes [41] (Table 4).

## 3. Computational Models for the Prediction of Cancer Metabolic Biomarkers

Single-cell sequencing allows for the study of the molecular changes occurring in individual cells within the tumor mass. Nonetheless, attributing a specific cellular annotation (in terms of cell type or metabolic state) is challenging, in particular to distinguish cancer cells in single-cell or spatial sequencing experiments. The information provided by high-throughput single-cell sequencing provides not only the description of distinct cellular annotations but also the functional annotation of single cells, for example the estimation of the differentiation potential, vulnerability to metabolic changes, and a prediction of cellular crosstalk [42]. However, the use of this technology also raises computational difficulties [43]. One of the major challenges in single-cell data analysis is to attribute a cell annotation to each cell analyzed [44]. The magnitude of the generated datasets renders the manual annotation processes unfeasible, whereas the peculiarities of data generation have stimulated the spread of novel and creative classification methods [45]. This limitation is particularly found in datasets coming from cancer tissues, in which the variability in the transcriptomic states does not conform to traditionally defined cell types [46,47].

In addition to the genome data, the transcriptome, proteome, and metabolome data offer snapshots of a cell’s phenotype space. As shown by PCAWG58 and TCGA59, which also provide transcriptome data in addition to genome data, the transcriptome, particularly RNA sequencing (RNA-seq), is the most frequently generated omics data among these. To perform more complex transcriptomic analyses, bulk RNA-seq has evolved into single-cell RNA-seq (scRNA-seq) and spatial RNA-seq. To enable a greater understanding of cell phenotypes, massive amounts of proteome and metabolome data are being generated for various human cells [48,49]. The Human Metabolome Database (HMDB) and Human Protein Atlas (HPA) are representative databases for the human proteome and metabolome, respectively. Integrative omics analysis has gained importance since these omics data are complementary to one another, and multiple omics data are frequently generated for a target cell [50,51].

Several studies have combined NGS data with ML to propose a novel data-driven methodology in systems biology [52]. Several network-based ML models have been implemented to analyze cancer data and aid in the understanding of novel mechanisms in cancer development [53,54]. Furthermore, the use of DNN models for large-scale data analysis enhanced the accuracy of computational models for the prediction of the mutational landscape, molecular subtyping and drug repurposing [55,56,57,58]. A growing number of DNN-based applications have recently integrated multi-omics and systems biology data into the learned models. Such approaches aim to apply the DNN model to well-established biomedical knowledge, thereby improving our understanding of diseases and therapeutic effects in novel ways [59,60].

A common aim of NGS data analysis in cancer research is the identification of potential biomarkers that are predictive of specific cancer types or subtypes. A variety of bioinformatics tools and ML models, for example, aim to identify a molecular signature that is significantly altered in cancer cells on a genomic, transcriptomic, or epigenomic level. Statistical and ML methods are typically used to identify the best set of biomarkers, such as single nucleotide polymorphisms (SNPs), mutations, or differentially expressed genes that are important in cancer progression. Previously, those markers had to be discovered or validated using time-consuming in vitro analysis. As a result, systems biology provides in silico solutions to validate such findings by utilizing biological pathways or gene ontology data [61].

## 4. AI in Cancer Prognosis

Detecting and predicting the course of the disease are key components to controlling tumor enlargement and providing adequate treatment to cancer patients. With the understanding that cancer can affect individuals differently, AI has been utilized to isolate subgroups within the patient population based on prognosis and survival data. Aside from segmentation, AI has pinpointed biomarkers that can indicate the recurrence of the disease. AI has been implemented to prognosticate high-risk neuroblastoma patients. Utilizing combined gene expression and copy number variations, an unsupervised learning algorithm called auto encoder determined significant features, which were then used for division into two clusters [62]. In a separate study, Francescatto et al. employed the integrative network fusion framework together with an ML classifier to distinguish features that could differentiate between distinct outcomes of patients [63].

DL-based neural networks have also been applied to breast cancer survival prognosis. To prevent overfitting effects due to the vast size of omics data, the SALMON survival analysis algorithm operates on eigengene matrices of co-expression network modules. To enhance robustness, it brings together traditional cancer biomarkers and multi-omics information and pinpoints key feature genes and cytobands [64]. The use of a DL-based algorithm allows for the combination of the information from the same gene across different types of omics data, thus resulting in a successful and insightful analysis [65].

## 5. AI in the Identification of Therapeutic Targets

A subset of alternative network approaches to identifying cancer targets are provided by network-based biology analysis algorithms. More importantly, because different algorithms can look at network data from different angles, they can compensate for each other to provide accurate biological explanations [66].

Interactome data can be organized and represented in the form of network structures to explain the molecular mechanisms underlying carcinogenesis, where the nodes are biological entities (genes, proteins, mRNAs, and metabolites) while the edges represent the associations–interactions between them (gene co-expression, signaling transduction, gene regulation, and physical interaction between proteins) [67,68]. AI algorithms could efficiently process biological network data by implementing classification, clustering, and prediction tasks in biological networks using machines or programs that enhance human intelligence [69]. As a result, AI algorithms will be able to elucidate the complexity of cancer behavior that rely on the interactions between genes and their products in biological network structures [70], allowing us to better understand carcinogenesis and identify novel anti-cancer targets [71].

One of the fundamental needs of precision oncology is anticipating therapy response for a patient population. The advantages of ML strategies have been tried for treatment response displaying and expectation following both center-based and component choice-based strategies [72]. The profound neural system-based examination has been used to predict therapy response. MOLI, a multi-omics late mix strategy in light of a profound neural system, consolidates somatic transformation, and duplicates number variation and quality articulation information to anticipate medication reaction conduct. MOLI is additionally utilized for board medication information, and information on medications with a similar target [73].

The Support Vector Machine (SVM) and the Leave-One-Out Cross-Validation (LOOCV) models have been employed to detect significant changes in RNA and miRNA transcriptomics data between from pancreatic ductal adenocarcinoma specimens and normal tissues. These features (selected RNAs and miRNAs) in combination with miRNA target expression data were further exploited to identify efficient diagnostic markers that were validated in other distinct datasets and biologically interpreted by pathway analysis of the corresponding target genes [74]. Moreover, ML-based analysis has been utilized to discover specific anticancer drug targets for breast tumors [75]. The characteristic genes extracted from multi-omics data of breast cancer with the aid of capsule network-based modeling were compared with well-known oncogenes, and novel genes were identified [76].

Recently, a comprehensive examination of nine cancers has demonstrated that proteomics data combined with gene expression, miRNAs expression and genomics is more effective in predicting the responsiveness of drugs and molecules specifically designed to target them. This research was conducted across 58 cell lines over nine cancers with Bayesian Efficient Multiple Kernel Learning (BEMKL) models [72]. This confirms the robustness of multi-omics data analysis across cancer types.

## 6. AI Clinical Application

The DELFI technology, which uses a blood test to indirectly evaluate the packing of DNA in the nucleus of a cell by assessing the bulk and amount of cell-free DNA present in the flow from various regions of the genome, is one example of AI in clinical practice. Cancer cells release DNA into the bloodstream when they die. DELFI uses ML to investigate millions of cell-free DNA pieces for unusual design in order to distinguish the occurrence of cancer. The strategy provides a perspective on cell-free DNA known as the “fragmentome” and only requires low-coverage genome sequencing, allowing the technology to be economically affordable in a screening setting [77].

The DELFI methodology finds that patients who were later diagnosed positive for cancer had a wide fluctuation in their fragmentome profiles, while those who had a negative cancer diagnosis had predictable fragmentome profiles. Overall, the technique was able to distinguish more than 90 percent of patients with lung cancer (including those with early stages) and displaying different subtypes [78].

Another study focused on glioblastoma, whose diagnosis is based on resection or biopsy which can be especially arduous and perilous in the case that the tumor mass is located in a deep position. Moreover, tracking cancer progression also necessitates repeated biopsies that are often impracticable. Consequently, there is an urgent requirement to identify biomarkers to diagnose and follow-up glioblastoma evolution by limiting the invasive approaches. Recently, an innovative cancer detection method has been developed based on plasma denaturation profiles obtained by a novel use of differential scanning fluorimetry. By comparing the denaturation profiles of blood samples collected from glioma patients and from healthy subjects, the researchers demonstrated that ML-based algorithms can automatically distinguish the cancer patients from the healthy individuals (with a precision around 92%). Additionally, this high-throughput workflow can be applied to any type of cancer and may represent a potent pan-cancer diagnostic and monitoring tool that requires only a plain blood test [79].

Among the limitations of the current approaches, tissue biopsy presents a fixed overview of the tumor that fails to record the intratumor distinguishment and dynamic changes occurring during carcinogenesis, also determined by clonal pressure caused by the applied medication [80]. On top of that, it is an invasive procedure, which usually cannot be performed multiple times on request, making this system unfeasible to be conducted as a regular practice for cancer patients’ long-term supervision and treatment adjustment. The emergence of liquid biopsy has been a revolutionary development for the current clinical practice, offering great potential to improve the management of ongoing cancer patients for the diagnosis, prognosis, and tailoring of treatment. This approach presents the advantage of being a minimally invasive procedure that utilizes tumor-derived materials obtained from several body fluids, such as peripheral blood, urine, pleural liquid, saliva, or ascites [81]. This solution is not limited by space or time, and it supplies clinically meaningful information related to both primary and metastatic malignant lesions. Among the components of tumor-derived materials that can be analyzed by liquid biopsy, circulating tumor cells, cell-free circulating nucleic acids, and extracellular vesicles are the most extensively studied and characterized cancer markers and are used for various objectives, for instance, the early detection of cancer, staging, prognosis, drug resistance, and minimal residual disease [82].

Another AI approach is the PinPoint test, a cost-effective AI-driven blood test for cancer that is meant to upgrade rapid cancer referral paths. The test is found on an algorithm that uses ML to investigate regular constituents, as well as the patient’s age and sex. It can calibrate and combine these individual variables into one solid and highly precise result, such as the likelihood that a patient has cancer [83]. The PinPoint test has been crafted as a decision support tool to give medical professionals the data they need to better sort patients when they initially present with symptoms. Those with high risk can be given precedence for speedy examination in secondary care, while those with the lowest risk can be securely excluded from the “2 week wait” pathway for further discussion with their physicians [84]. This strategy of pinpointing those at the greatest risk for prioritization will promote early detection, contribute to a more dependable pathway, and assist in decreasing post-pandemic delays [85].

## 7. AI imaging in Cancer Diagnosis

In the field of cancer imaging, AI displays a great utility in three main clinical tasks: tumor detection, characterization, and monitoring [86]. The localization of objects of interest in radiographs is referred to as detection, and it is a subset of computer-aided detection (CADe). AI-based detection tools can be used to reduce observational errors and serve as a first line of defense against omission errors [87].

Characterization in general includes tumor segmentation, diagnosis, and staging. It can also include a disease-specific prognosis as well as outcome prediction based on specific treatment modalities. Segmentation determines the extent of abnormalities and can range from simple 2D measurements of the maximum in plane tumor diameter to more involved volumetric segmentations that assess the entire tumor as well as any surrounding tissues. This information could be exploited for future diagnostic purposes as well as for calculating the appropriate dose administration during radiation planning. AI has the capability to significantly improve the efficiency, reproducibility, and reliability of tumor measurements through automated segmentation. In computer-aided diagnosis (CADx) systems, systematic processing of quantitative tumor features is used, allowing for more reproducible descriptors. In the case of inconsistencies in interpretation by different human readers, CADx systems have been used to diagnose lung nodules in thin-section CT and prostate lesions in multiparametric MRI [88].

Staging is another aspect of tumor characterization in which tumors are classified into predefined groups based on the size and spread of the tumor mass, thus providing information regarding the expected clinical course and for the decision of the most appropriate treatment strategies [89]. The application of AI-based methods to cancer imaging allows for the estimation of tumor size, shape, morphology, texture, and kinetics. Additionally, the use of dynamic assessment of contrast uptake on MRI enables physicians to characterize the tumor mass in terms of heterogeneity, phenotypes of spatial features and dynamic characteristics [90]. Another variable taken in consideration from AI-based tools is entropy, a mathematical descriptor of randomness that provides information on how heterogeneous the pattern is within the tumor, thereby describing the heterogeneous pattern of vascular system uptake (contrast uptake) within tumors imaged on contrast-enhanced breast MRI. As demonstrated by the NCI’s The Cancer Genome Atlas (TCGA) breast cancer dataset, such analyses could reflect the heterogeneous nature of angiogenesis and treatment susceptibility [91].

DL systems have been used to simultaneously detect and classify prostate lesions. For training convolutional neural networks (CNNs) for prostate cancer diagnosis by MRI, both de novo training [92] and the transfer learning of pre-trained models [93] have been successful. The implementation of CNNs models with anatomically aware features has been shown to improve their performance [94,95]. In addition to MRI, AI techniques for prostate cancer classification have shown promising results by integrating ultrasound data, specifically radiofrequency. Again, both traditional ML and DL approaches were used to train classifiers to estimate the grading of prostate cancer by exploiting temporal ultrasound data [96].

## 8. Critical Issues, Challenges, and Limitations

The accuracy and consistency of AI systems are frequently restricted by their training data and the hardware used. We must keep in mind that AI can make mistakes in some situations because its decision-making ability is predictive and probabilistic. As a result, there are no clear regulations or guidelines in place to determine who is legally liable when AI malfunctions occur or causes issues while providing a service. Another factor to take in consideration is that most of the places where the potential of AI in healthcare has been evaluated are basically high-income and resource-driven areas. When used in low-income countries with a shortage of well-trained physicians and oncological specialists, AI-based prediction tools are expected to have a greater impact and increment the success of cancer treatment.

The improvement in the AI interpretation is a crucial step toward mitigating this risk and providing a decision-making rationale. One limitation is represented by the lack of a human verification step in the process unless a physician supervises the AI system. As a result, no one expects AI to entirely replace medical professionals. AI-based precision medicine will be critical for cancer treatment in the future. Living databases will exploit extremely complex models capable of making a personalized therapy selection, estimation of the drug dose, follow-up schedule, and so on. However, the transition from artificial narrow intelligence to artificial general intelligence will result in the automation of all the steps involved in cancer prediction, diagnosis, and treatment.

Despite its numerous benefits, AI presents several challenges and constraints that hinder it from fully functioning in cancer research. Particularly, three layers of complexity must be considered: (i) cancer is a highly heterogeneous organoid-like structure that, at the time of diagnosis, is made up of many different cancer subclones embedded in a stroma (the tumor microenvironment) that itself contributes to cancer progression; (ii) as cancer progresses, tumor evolution leads to increased intratumor heterogeneity so that by the time therapy is started, the targeted cancer may not respond; (iii) cancers with the same molecular and histological signatures behave differently in each single patient because of individual epigenetic and immunological modulations [97,98,99]. Thus, the final clinical outcome will depend on the complex interplay between the cancer (with its multiple subclones) and the tumor microenvironment (which includes the stroma composition and the inflammatory and immune response), and, finally, the general pathophysiological condition of the patient (e.g., the body mass, the adipose tissue mass, the nutrition status, the psychological status, the immune status, etc.). This poses an important limit to the capability of AI in predicting the therapy efficacy and the prognosis, which once again stresses the fundamental role of the clinician that cannot be substituted by an algorithm.

The new era of innovation brings with it many challenges that should be overcome to drastically improve oncology procedures at several levels. The lack of inclusive and different datasets for training represents a significant obstacle to the widespread adoption of AI algorithms and decision-support systems in cancer care. Most of the powerful AI models require a large sample size to efficiently train the tool. Although there are dimensionality reduction and feature selection methods for addressing these aspects, proper implementation is critical for achieving better and reliable results. The number and type of data annotated influences the constructions of algorithms, and an imbalance in data from patients differing for gender, age, race, nutritional state, lifestyle, and environment will affect AI and ML training. Thus, the lack of sensible data may increase the risk of missed diagnosis. Therefore, experts are fundamental in data curation and data annotation to provide reliable datasets to be used for training AI classifier and predictors models.

In medical data sets, particularly in the case of cancer data, classes are typically distributed unequally. The continuous use of AI- and ML-based tools for diagnosis and treatment decisions can be risky due to distributional shifts, which means that target data may not match the ongoing patient data employed to train the model, resulting in incorrect outputs. Predictions made by AI at the time of diagnosis likely changes during the course of the therapy and the evolution of the disease along with changes in patient’s habit (style of life, diet, medications, etc.).

Changes in technology, healthcare, and population, such as the gene pool, are likely to have an impact on the relationship between the data items. The actual application of AI models in clinics is not being actively considered. The predictions achieved with these models frequently require validation in the clinical practice to assist medical experts in confirming diagnosis decisions.

Significant issues regarding data availability and interpretability caused by AI’s “black box” process, in parallel with the emergence of an inherent bias toward limited cohorts that reduces the reproducibility of AI models and perpetuates disparities in the healthcare, collectively prevent the widespread application of AI in clinics. Additionally, the distribution of AI-based technologies in many developing countries may be hampered by a lack of knowledge in computing algorithms and technologies of the physicians.

Taken together, the clinically relevant achievements discussed in the present review need to become more solid to be translated into the right treatment for the right patient. Hence, the rapidly ongoing evolution of AI-based medical data analysis will significantly improve the treatments in cancer.

## 9. Conclusions and Perspectives

**Figure 3:**
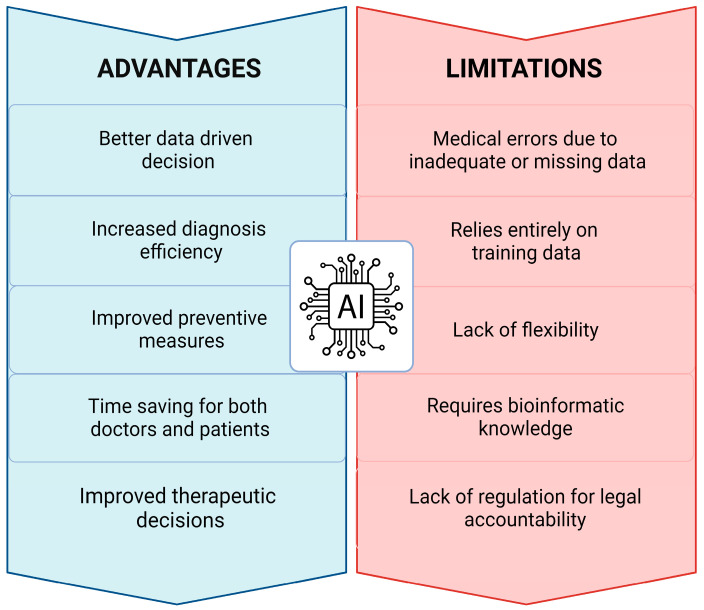
Advantages and limitations of AI. The scheme summarizes the main benefits along with the current concerns related to the use of AI in the clinical practice.

In this paper, we present an overview of the models applied in diagnosing and identifying therapeutic targets, and we discussed the challenges and future perspectives of AI in cancer research (Figure 3). As the power and potential of AI are increasingly demonstrated, in the coming future several other biomedical fields may exploit the use of AI in their routine clinical practice. AI methodologies’ accuracy and predictive power must be significantly improved, as well as demonstrated efficacy comparable to, or better than, human experts in controlled studies [100]. Up to now, AI shows early promising results in the management of several disease conditions, but more efforts in prospective trials and in the education of physicians, technologists, and physicists are needed before it can be widely used. Although there will always be a “black box” for human experts to view AI-generated results, data visualization tools are becoming more widely available to provide some visual understanding of how algorithms make decisions [101]. It is to be stressed that AI is meant to complement the medical doctor facilitating his work, but it will not replace the medical doctor.

## Figures and Tables

**Table 1:** Summary of the main advantages and limitations of PLS models.

Advantages	Limitations
Ability to robustly handle more descriptor variables	Higher risk of overlooking ‘real’ correlations
Provide more predictive accuracy	Sensitivity to the relative scaling of the descriptor variables
Low risk of chance correlation	

**Table 2:** Summary of the main advantages and limitations of GEM models.

Advantages	Limitations
Explore metabolism in multiple cell types	Uncertainties in the estimated parameters regarding quantitative flux predictions
Validating or discovering biomarkers for screening, diagnostics, prognostics, and/or patient stratification	Ambiguous normalization of experimentally quantified fluxes
Identify cancer-specific metabolic features that constitute generic potential drug targets for cancer treatment	

**Table 3:** Summary of the main advantages and limitations of DNN models.

Advantages	Limitations
Ability to handle complex data and relationships	Massive data requirement
Effective at producing high-quality results	High processing and computational power
Extremely scalable because of its capacity to analyze large volumes of data	Black box problem making them hard to debug and understand how they make decisions

**Table 4:** Summary of the main advantages and limitations of GNN models.

Advantages	Limitations
Rapid processing of massive data	Limited to a fixed number of points
Reliable performance in mining deep-level topological information	Time and space complexity are higher
Extracting text relationship and reasoning the structure of graphics and images	Less handling of edges of graphs based on their types and relations

## Data Availability

No new data were created or analyzed in this study. Data sharing is not applicable to this article.

## Abbreviations

Artificial Intelligence, AI; Bayesian Efficient Multiple Kernel Learning, BEMKL; computed tomography, CT; computer-aided detection, CADe; computer-aided diagnosis, CADx; convolutional neural networks, CNNs; deep learning, DL; deep neural network, DNN; gene expression modeling, GEM; graph convolutional network, GCN; graph neural networks, GNNs; Human Metabolome Database, HMDB; Human Protein Atlas, (HPA); Leave-One-Out Cross-Validation, LOOCV; machine learning, ML; magnetic resonance imaging, MRI; nano differential scanning fluorimetry, Nanodsf; next-generation sequencing, NGS; positron emission tomography, PET; partial least squares-discriminant analysis, PLS-DA; single nucleotide polymorphisms, SNPs; single-cell RNA sequencing, scRNA-seq; Support Vector Machine, SVM; The Cancer Genome Atlas, TCGA; ultrasound imaging, US.
